# Urban-rural disparities of antenatal care in South East Asia: a case study in the Philippines and Indonesia

**DOI:** 10.1186/s12889-021-11318-2

**Published:** 2021-06-24

**Authors:** Ratna Dwi Wulandari, Agung Dwi Laksono, Nikmatur Rohmah

**Affiliations:** 1 Faculty of Public Health, Universitas Airlangga Surabaya, Campus C Mulyorejo, Surabaya, 60115 Indonesia; 2 National Institute of Health Research and Development of The Ministry of Health of the Republic of Indonesia, Percetakan Negara 29, Jakarta, 10560 Indonesia; 3 Faculty of Health Science, Muhammadiyah University of Jember, Gumuk Kerang, Karangrejo, Kec. Sumbersari, Jawa Timur, Kabupaten Jember, East Java 68124 Indonesia

**Keywords:** Urban-rural, Disparity, Antenatal care, Mother and child health, Southeast Asia

## Abstract

**Background:**

The government is obliged to guarantee equal access to antenatal care (ANC) between urban and rural areas. This study aimed to analyze urban-rural disparities in ≥4 ANC visits during pregnancy in the Philippines and Indonesia.

**Methods:**

The study processed data from the 2017 PDHS and the 2017 IDHS. The analysis unit was women aged 15–49 years old who had given birth in the last 5 years. The weighted sample size was 7992 respondents in the Philippines and 14,568 respondents in Indonesia. Apart from ANC as the dependent variable, other variables analyzed were residence, age, husband/partner, education, parity, and wealth. Determination of urban-rural disparities using binary logistic regression.

**Results:**

The results show that women in the urban Philippines are 0.932 times more likely than women in the rural Philippines to make ≥4 ANC visits. On the other side, women in urban Indonesia are more likely 1.255 times than women in rural Indonesia to make ≥4 ANC visits. Apart from the type of residence place (urban-rural), five other tested multivariate variables also proved significant contributions to ANC’s use in both countries, i.e., age, have a husband/partner, education, parity, and wealth status.

**Conclusions:**

The study concluded that disparities exist between urban and rural areas utilizing ANC in the Philippines and Indonesia. Pregnant women in the rural Philippines have a better chance of making ≥4 ANC visits. Meanwhile, pregnant women in urban Indonesia have a better chance of making ≥4 ANC visits.

## Background

The high Maternal Mortality Rate (MMR) in several world regions reflects inequality in access to quality health services. Indonesia is among the countries with the third-highest MMR in Southeast Asian countries after Myanmar and Laos [1, 2]. The MMR in Indonesia in 2017 was 177 deaths per 100,000 live births. The MMR in Indonesia has gradually decreased from 207 per 100,000 live births in 2013 to 177 per 100,000 live births in 2017 [3]. The high MMR also applies to the Philippines. The maternal mortality ratio in the Philippines stood at 121 deaths per 100,000 live births in 2017 to 124 deaths per 100,000 live births in the previous year. Data in Indonesia and the Philippines show a gradual downward trend but have not yet reached the SDGs target of less than 70 per 100,000 live births [4, 5].

Singapore, Malaysia, Brunei, Thailand, and Vietnam are Southeast Asia Countries with MMR that have achieved the SDGs target. The five countries from 2013 to 2017 showed a downward trend, except for Brunei Darussalam. Singapore, Malaysia, Brunei, Thailand, and Vietnam in 2017 were at number 8; 29; 31; 37, and 43 per 100,000 live births [4]. This data provides information that MMR in Indonesia is 6.1 times higher than in Malaysia and 22 times higher than in Singapore. Meanwhile, MMR in the Philippines was 3.9 times higher than Brunei Darussalam, even 15.1 times higher than Singapore. With an average decline in 3–6% MMR every year, the Philippines and Indonesia still strive to achieve the SDGs target [4].

The causes of maternal death can be either direct or indirect. The leading natural causes are bleeding, hypertension in pregnancy, sepsis, complications of childbirth [6, 7]. Bleeding and pre-eclampsia/eclampsia accounted for 43.4 and 36.9% of maternal death in West Nigeria. Similar to the results of previous studies, it stated that the direct causes of death for maternal in South Africa contributed more than two times higher than the indirect causes of death [8]. Maternal death indirect reasons include age, inadequate human resources, delays in seeking treatment, inadequate equipment, obstacles to transportation and delays in referring, and insufficient antenatal care (ANC) [8, 9]. Low ANC visits and unequal access to antenatal services are indirect causes of maternal death that need serious attention [10]. We could prevent and saved most the maternal death. Mothers who should not have died eventually died because they did not get adequate prevention and treatment efforts [1].

ANC is used to detect and prevent direct and indirect causes of maternal death [1]. Although the 2016 WHO guidelines for ANC shift the recommended minimum number of ANC contacts from four to eight, the Indonesian government still uses the basic ANC model, which includes four ANC visits between 8 and 12 weeks of gestation, between 24 and 26 weeks, at 32 weeks, and between 36 and 38 weeks [11].

Several previous studies have suggested disparities in the ANC. A prior study in Indonesia with Papua referenced the gaps in antenatal care services in all regions except Maluku [12]. The socioeconomic level also has a role in increasing ANC visits and the involvement of husbands in ANC. The better the socioeconomic status of women in urban areas, the more likely it is to have antenatal visits and the more likely it is that their husbands will be involved in ANC [13, 14]. On the other hand, previous studies in Indonesia, Ethiopia, and Nigeria found evidence that women who live in urban areas have a greater chance of doing ANC at least four times than women in rural areas [15–17].

Moreover, women in urban areas who do ANC at least four times are more likely to give birth in health facilities than women in rural areas [18, 19]. Adequate ANC visits are an effort to detect possible early complications during pregnancy. We expected pregnant women who carry out ANC regularly to receive sufficient information about pregnancy complications [20].

We believed ANC to be an opportunity to promote care skills at delivery and healthy behavior after the puerperium. These behaviors include breastfeeding skills, puerperal care, and planning for optimal pregnancy spacing [21]. ANC has a beneficial effect on the next generation’s health or for the child to be born. Moreover, ANC improves mothers’ and children’s health and reduces maternal mortality [22, 23]. This study aimed to analyze urban-rural disparities in ≥4 ANC visits during pregnancy in the Philippines and Indonesia based on the background description.

## Methods

### Data source

The author conducted the study using secondary data from the 2017 Philippine Demographic Health Survey (PDHS) and the 2017 Indonesian Demographic Health Survey (IDHS). The PDHS and the IDHS were part of the international Demographic and Health Survey (DHS) program conducted by the Inner City Fund (ICF). The study takes samples through stratification and multistage random sampling methods.

The Master Sample Frame (MSF) prepared and produced by the Philippine Statistics Authority was utilized as the sample frame for the 2017 PDHS (PSA). The Philippines’ survey splits into 17 administrative regions, each of which is further subdivided into provinces, highly urbanized cities (HUC), and other particular areas. There are 81 provinces in the Philippines, 33 HUCs, and three more special zones. There are 42,036 barangays in the Philippines, with 5697 of them being urban and the rest being rural. The MSF’s main sampling units (PSUs) were created based on the 2010 Census of Population and Housing (CPH) findings and were revised in August 2015 based on the 2015 Census of Population results. The PSUs were reconstructed using the 2015 Enumeration Areas Reference File (EARF), and the 2015 lists of dwelling units served as the Secondary Sampling Unit (SSU) frame. A PSU can be a barangay, a section of a larger barangay, or two or more minor barangays nearby. Out of 42,036 barangays, there are a total of 87,098 PSUs (910 barangays were reported as least accessible and were excluded from the MSF) [24].

Meanwhile, the sampling design used in the 2017 IDHS is stratified two-stage sampling, namely: Stage 1, selecting several census blocks in a systematic proportional to size probability with the size of the number of households resulting from the 2010 population census listing. In this example, an implicit stratification procedure based on urban and rural regions was used and sorting census blocks based on the wealth index category of the 2010 population census data. Stage 2 picks 25 ordinary households in each census block based on updating the households in each census block [25].

In this study, the analysis unit was women aged 15–49 years old who had given birth in the last 5 years. The study obtained a weighted sample size of 7992 respondents in the Philippines and 14,568 respondents in Indonesia using the unit analysis criteria.

### Variables

Although the 2016 WHO guidelines for ANC shift the recommended minimum number of ANC contacts from four to eight, the Indonesian and Philippines government still uses the basic ANC model, includes four ANC visits between 8 and 12 weeks of gestation, between 24 and 26 weeks, at 32 weeks, and between 36 and 38 weeks [11]. Based on these policies, this study divides the ANC into two categories, namely < 4 ANC visits and ≥ 4 ANC visits. Other variables analyzed as independent variables were the type of place of residence, age group, have a husband/partner, education level, parity, and wealth status.

The type of place of residence consists of two categories, namely urban and rural. This categorization refers to the Philippine Statistics Authority and Statistics Indonesia. The age group consists of seven types in 5 years, namely 15–19, 20–24, 25–29, 30–34, 35–39, 40–45, and 45–49. Have a husband/partner consists of two categories, namely not having and having. Education level was the respondent’s recognition of the last diploma they have—education level consists of four categories: no education, primary, secondary, and higher. Parity was the number of living children that have been born. Parity consists of three types, namely primiparous (< 1), multiparous (2–4), and grand multiparous (> 4).

The study determined wealth status based on the wealth quintile owned by a household. Households were scored based on the numbers and types of items they had, from televisions to bicycles or cars, and housing characteristics, such as drinking water sources, toilet facilities, and primary building materials for the house’s floor. The study calculated the score using principal component analysis. National wealth quintiles were arranged based on household scores for each person in the household and then divided by the distribution into the same five categories, accounting for 20% of the population. Wealth status consists of five classes: the poorest, poorer, middle, richer, and the richest [26].

### Data analysis

In the first stage, the study employed chi-square to analyze urban-rural characteristics and other variables at the initial stage. In the final step, because of the dependent variable’s nature, binary logistic regression was used to determine the odds ratio with a 95% confidence interval (CI). The study used SPSS 21 software for all stages of statistical analysis.

### Ethical approval

The 2017 PDHS and the 2017 IDHS have obtained ethical clearance from the National Ethics Committee in the Philippines and Indonesia. The study deleted all respondents’ identities from the dataset. Respondents have provided written approval for their involvement in the research. The researcher has obtained permission to use the 2017 IDHS and the 2017 PDHS data through the website: https://dhsprogram.com/ for this study.

## Results

### Bivariate analysis

Table 1 presents the bivariate analysis results between residence type (urban-rural) and other variables. Based on the ANC frequency, Indonesia, both in urban and rural areas, has ≥4 ANC visits higher than that in the Philippines. The two countries tend to be mainly populated by the 25–29 and 30–34 age groups based on the age group. Based on having a husband/partner in the Philippines, more women have a husband/partner in rural areas than in urban areas. Meanwhile, in Indonesia, the percentage of women with a husband/partner appears to be more balanced between urban and rural areas.

**Table 1:** The results of bivariate analysis between the type of place of residence (urban-rural) and other variables of respondents in the Philippines (*n* = 7992) and Indonesia (*n* = 14,568) in 2017

Variables	Philippine (***n*** = 7992)			Indonesia (***n*** = 14,568)		
	Urban (2602)	Rural (5390)	p	Urban (7322)	Rural (7246)	p
ANC			***0.000			***0.000
• < 4	11.3%	15.0%		3.9%	7.3%	
• ≥ 4	88.7%	85.0%		96.1%	92.7%	
Age group			***0.000			***0.000
• 15–19	3.9%	4.9%		1.9%	2.9%	
• 20–24	19.9%	21.1%		13.9%	19.2%	
• 25–29	27.8%	25.2%		25.0%	25.6%	
• 30–34	22.4%	20.8%		26.8%	24.6%	
• 35–39	15.4%	17.4%		21.8%	18.4%	
• 40–44	8.6%	8.3%		8.8%	7.5%	
• 45–49	1.9%	2.2%		1.7%	1.8%	
Have a husband/partner			***0.000			***0.000
• No	10.2%	6.5%		2.9%	2.8%	
• Yes	89.8%	93.5%		97.1%	97.2%	
Education Level			***0.000			***0.000
• No education	0.4%	1.4%		0.4%	1.0%	
• Primary	11.4%	20.2%		17.5%	32.5%	
• Secondary	52.6%	50.6%		62.6%	55.8%	
• Higher	35.6%	27.9%		19.6%	10.8%	
Parity			***0.000			***0.000
• Primiparous	31.7%	27.0%		33.7%	34.0%	
• Multiparous	57.8%	55.1%		62.6%	60.3%	
• Grand multiparous	10.5%	17.9%		3.7%	5.7%	
Wealth status			***0.000			***0.000
• Poorest	12.3%	35.5%		6.6%	29.9%	
• Poorer	16.7%	26.1%		13.4%	26.7%	
• Middle	25.3%	16.4%		21.1%	20.8%	
• Richer	24.1%	12.6%		27.3%	14.9%	
• Richest	21.6%	9.3%		31.6%	7.8%	

^∗^*p* < 0.05; ^∗∗^*p* < 0.01; ^∗∗∗^*p* < 0.001

According to the education level, both countries are health by large by women with secondary education levels, both urban and rural. Based on parity, the two countries are more by multiparous women in urban and rural areas. Finally, based on wealth status, in rural areas, the poorest women rule both countries. Meanwhile, the richest women lead urban Indonesia, and women who have a middle-class wealth status break the urban Philippines.

**Fig. 1:**
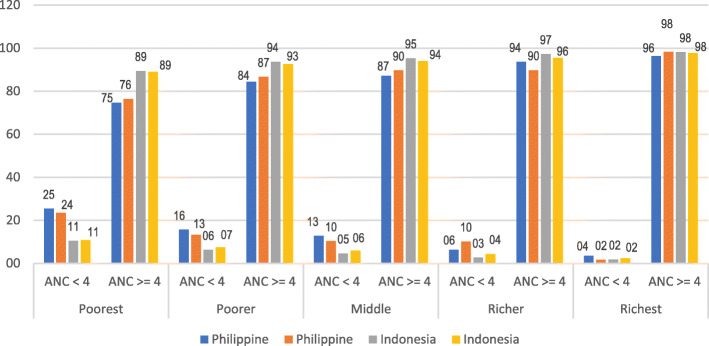
The interaction diagram between the type of place of residence, frequency of ANC, and wealth status in the Philippines (*n* = 7992) and Indonesia (*n* = 14,568) in 2017

Figure 1 shows a diagram of the interaction between three variables: residence, frequency of ANC, and wealth status in the Philippines and Indonesia. In the wealthiest group, both countries show that women who make ≥4 ANC visits tend to be more influential than other wealth status groups, both in urban and rural areas. On the other hand, in the most inferior group, Indonesian women who make ≥4 ANC visits tend to be better than Philippine women.

### Multivariable analysis

Table 2 displays the results of binary logistic regression of ANC in the Philippines and Indonesia. The multivariable analysis used “<4 ANC visits” as reference. Women in the urban Philippines are 0.932 times more likely than women in the rural Philippines to make ≥4 ANC visits (AOR 0.932; 95% CI 0.932–0.932). On the other side, women in urban Indonesia were 1.255 times more likely than women in rural Indonesia to make ≥4 ANC visits (AOR 1.255; 95% CI 1.255; 95% CI 1.255–1.255). This analysis shows that in the Philippines, women who live in rural areas are more likely to make ≥4 ANC visits. Meanwhile, in Indonesia, women living in urban areas have a better chance of making ≥4 ANC visits.

**Table 2:** The results of binary logistic regression of ANC in the Philippines (*n* = 7992) and Indonesia (*n* = 14,568) in 2017

Variables	Philippine			Indonesia		
	≥ 4 ANC Visits			≥ 4 ANC Visits		
	AOR	95% CI		AOR	95% CI	
		LB	UB		LB	UB
Type of place of residence						
• Urban	***0.932	0.932	0.932	***1.255	1.255	1.255
• Rural	–	–	–	–	–	–
Age group						
• 15–19	–	–	–	–	–	–
• 20–24	***1.359	1.359	1.359	***1.991	1.991	1.992
• 25–29	***2.096	2.095	2.096	***2.756	2.755	2.758
• 30–34	***2.620	2.619	2.621	***2.929	2.928	2.930
• 35–39	***2.185	2.184	2.186	***3.082	3.081	3.083
• 40–44	***2.159	2.159	2.160	***3.255	3.253	3.256
• 45–49	***1.976	1.975	1.977	***5.383	5.379	5.387
Have a husband/partner						
• No	–	–	–	–	–	–
• Yes	***1.928	1.928	1.929	***1.777	1.777	1.778
Education Level						
• No education	–	–	–	–	–	–
• Primary	***2.464	2.463	2.466	***2.169	2.168	2.170
• Secondary	***4.116	4.114	4.118	***2.534	2.532	2.535
• Higher	***6.522	6.519	6.526	***2.745	2.743	2.746
Parity						
• Primiparous	–	–	–	–	–	–
• Multiparous	***0.705	0.705	0.705	***0.646	0.645	0.646
• Grand multiparous	***0.406	0.406	0.406	***0.249	0.249	0.249
Wealth status						
• Poorest	–	–	–	–	–	–
• Poorer	***1.518	1.518	1.519	***1.363	1.362	1.363
• Middle	***1.594	1.594	1.594	***1.660	1.659	1.660
• Richer	***2.098	2.098	2.099	***2.465	2.464	2.466
• Richest	***5.275	5.273	5.277	***3.925	3.924	3.927

95% CI; ^∗^*p* < 0.05; ^∗∗^*p* < 0.01; ^∗∗∗^*p* < 0.001; *AOR* adjusted odds ratio, *LB* lower bound, *UB* upper bound

Apart from the residence place (urban-rural), five other tested multivariate variables also proved significant contributions to ANC’s use in both countries. Indonesia’s age group shows that the older it is, the more it makes ≥4 ANC visits. Conditions are different in the Philippines, which does not show any particular trend. Women in the 30–34 age group dominate the use of ≥4 ANC visits in the Philippines.

According to have a husband/partner, women in both countries with a husband/partner have a higher probability of making ≥4 ANC visits. The trend is that Philippine women have a higher likelihood than Indonesian women. Based on the education level, the two countries have the same tendency. The higher the level of education a woman has, the higher the likelihood of making ≥4 ANC visits. The effect of the education level on the use of ≥4 ANC visits was stronger for Philippine women than for Indonesian women.

Based on parity, both countries have the same tendency. The higher the parity, the lower the chances of a pregnant woman having ≥4 ANC visits. This information suggests that primiparous women in both countries have a higher likelihood of having ≥4 ANC visits. Finally, based on wealth status, both countries also have the same tendency. The better the wealth status, the higher the chance for pregnant women to have ≥4 ANC visits. The effect of wealth status on the use of ANC tends to be healthier for Philippine women than for Indonesian women.

## Discussion

In general, based on the memories of women who did ANC, the percentage of the types of services they received was relatively high. In the Philippines, blood pressure is measured: 99.1% in an urban area and 98.1% in a rural area; weight measured: 99.3% in an urban area and 98.0% in a rural area; height measured: 89.5 in an urban area and 84.8 in a rural area [24]. Meanwhile, in Indonesia, blood pressure measured: 98.9% in an urban area and 97.4% in a rural area; weight measured: 98.7% in an urban area and 96.2% in a rural area; height measured: 70.2 in an urban area and 67.6 in a rural area [27]. This information shows that ANC services in the Philippines are slightly better than the ANC services in Indonesia.

The analysis in this study found that in the Philippines, women living in rural areas were more likely to make ≥4 ANC visits. Meanwhile, in Indonesia, women living in urban areas have a better chance of making ≥4 ANC visits. The disparity between urban and rural areas often occurs due to development inequality between the two categories of places. Several studies often found that those who live in urban areas have access to better health services. This condition is related to better availability in urban areas [18, 28–30]. Previous studies in Nigeria, Angola, Bangladesh, and Tanzania also informed that the type of residence affected the use of ANCs [31–34].

The Philippines’ phenomenon of findings informs that pregnant women in rural areas are more likely to have ≥4 ANC visits than results in various countries [31–33]. Although the odds ratio is not too big, close to 1 (AOR 0.932), it is interesting to study this phenomenon in the Philippines. The situation shows the context of health development between urban and rural areas in the Philippines, different from other countries. Another possibility is the existence of specific programs or policies related to the ANC enforced in the Philippines.

The Philippine government has a specific financing policy in both areas. The Philippine government provides incentives in the form of free maternal services and cash grants. This policy is considered successful in encouraging mothers to go to health facilities for ANC and delivery at the facility. Free services are provided by PhilHealth (state social health insurance), while they offer cash grants through government and other community partners’ conditional cash transfer programs. The government provided pregnant women with financial risk protection through this financial incentive. The policy has been shown to increase ANC visits as well as make a balance between the two areas.

On the other hand, there is also a disincentive policy in the form of a local regulation prohibiting childbirth at home. The penalties include fines for mothers and birth attendants if they found the mother giving birth outside of a health facility [35]. Moreover, several parties in the Philippines’ efforts to mobilize voluntary community participation have contributed to encouraging maternal services in health facilities. A study in the Philippines reports that Ayods’ involvement in tracking and supporting pregnant women appears to have some successful health outcomes. This pattern employs community health workers and volunteers, creating a sustainable model for isolated communities, especially in rural areas [36].

Moreover, a previous study found that disparities between urban and rural areas in Indonesia occurred in all regions. The largest island grouped the division of regions in Indonesia [12]. The study found age has influenced the use of ANC in both countries. Perhaps this situation relates to the maturity of a woman in managing risks during pregnancy. The older, it felt the more the experience and the ability to make better judgments [37, 38]**.** Two studies found a similar research conduct in Bangladesh and sub-Saharan Africa [39, 40]. Meanwhile, a study in Rwanda informed that low ANC services’ risk was higher among women aged 31 years or older [41].

Women in both countries who have a husband/partner are more likely to have ≥4 ANC visits. Previous research in Rwanda, Ghana, India, and Lao has informed that prove husband/partner support increases ANC utilization [41–44]. The risk of low utilization of ANC services was higher among single women, with an odds ratio of almost three times [41]. Pregnancy is a collaborative process between women and men. As a husband and partner, men’s presence increases awareness of a pregnant woman in her pregnancy. Women have a place to share the burden, both psychologically and financially [45].

The higher the level of education a woman has, the higher the likelihood of making ≥4 ANC visits. The results of previous studies inform that a better level of education is proven to increase a woman’s independence to decide what is best for her. A better education class also provides a better understanding of each risk of the chosen action course [46]. A previous study showed better education influences women’s knowledge of pregnancy’s danger signs [47]. Moreover, education is again proven to play a role in a person’s perception of health services quality [48, 49]. Several previous studies also found results in line with this research in various countries, including Kenya, Bangladesh, and Ethiopia [50–52]. Several other lessons also inform that education is a robust positive determinant of boosting performance in the health sector [3, 53, 54]. On the other hand, several studies reported poor education as a barrier to achieving quality performance in the health sector [55, 56].

The results found that primiparous women in both countries had a higher likelihood of having ≥4 ANC visits. The possibility of this is closely related to the caution of women who have experienced pregnancy for the first time or the minimal experience of women who have only had one child. However, multiparous/grand multiparous women have a higher risk of pregnancy hazards than primiparous women [57]. Several previous studies in various countries also found parity as a determinant of ANC utilization [58–60]. The results are consistent but from a different perspective, informed in a study in Ghana. Higher parity was significantly associated with low utilization of ANC [61].

The analysis also found that the better the wealth status, the higher the probability of pregnant women having ≥4 ANC visits. Previous research has suggested that women with low wealth status may have a cost barrier to accessing ANC services during pregnancy [13, 62]. Previous research on ANC’s use in Pakistan, Nigeria, and Uganda informed that wealth status has a positive relationship with ANC visits. Women with no cost problems have a better chance of utilizing ANC [63–66].

The results of this study are considered helpful for policy-makers in both countries. The results of the analysis show that urban-rural disparities still exist in both countries. A focused policy is needed if we want to reduce this disparity. The policy objectives to be intervened must be specific. Based on this study’s analysis results, the particular targets are younger women, women who do not have a husband/partner, poor education women, women with many children, and low wealth status.

### Study limitation

This study has limitations as a consequence of the use of secondary data received. This study does not analyze cultural factors and beliefs known in previous studies to influence ANC utilization [67–69].

## Conclusions

Based on the research results, the study concluded that disparities exist between urban and rural areas that apply to ANC in the Philippines and Indonesia. Pregnant women in the rural Philippines have a better chance of making ≥4 ANC visits. Meanwhile, pregnant women in urban Indonesia have a better chance of making ≥4 ANC visits. Meanwhile, apart from the residence type (urban-rural), five other variables tested were also proven to significantly contribute to ANC’s use in both countries, namely age group, husband/partner, education level, parity, and wealth status.

## Data Availability

The authors cannot share data because a third party and authors who own the data do not have permission to share it. The 2017 IDHS data set name requested from the ICF (‘data set of childbearing age women’) is available from the ICF contact https://dhsprogram.com/ for researchers who meet the access criteria to confidential data.

## Abbreviations

ANC: Antenatal Care
IDHS: Indonesia Demographic and Health Survey
ICF: Inner City Fund
AOR: Adjusted odds ratio
LB: Lower bound
UB: Upper bound
